# Supplementary figures and images for: Gait changes in a line of mice artificially selected for longer limbs
Source: PeerJ. 2017 Feb 22;5:e3008. doi: 10.7717/peerj.3008 (PMC5324776; doi:10.7717/peerj.3008)

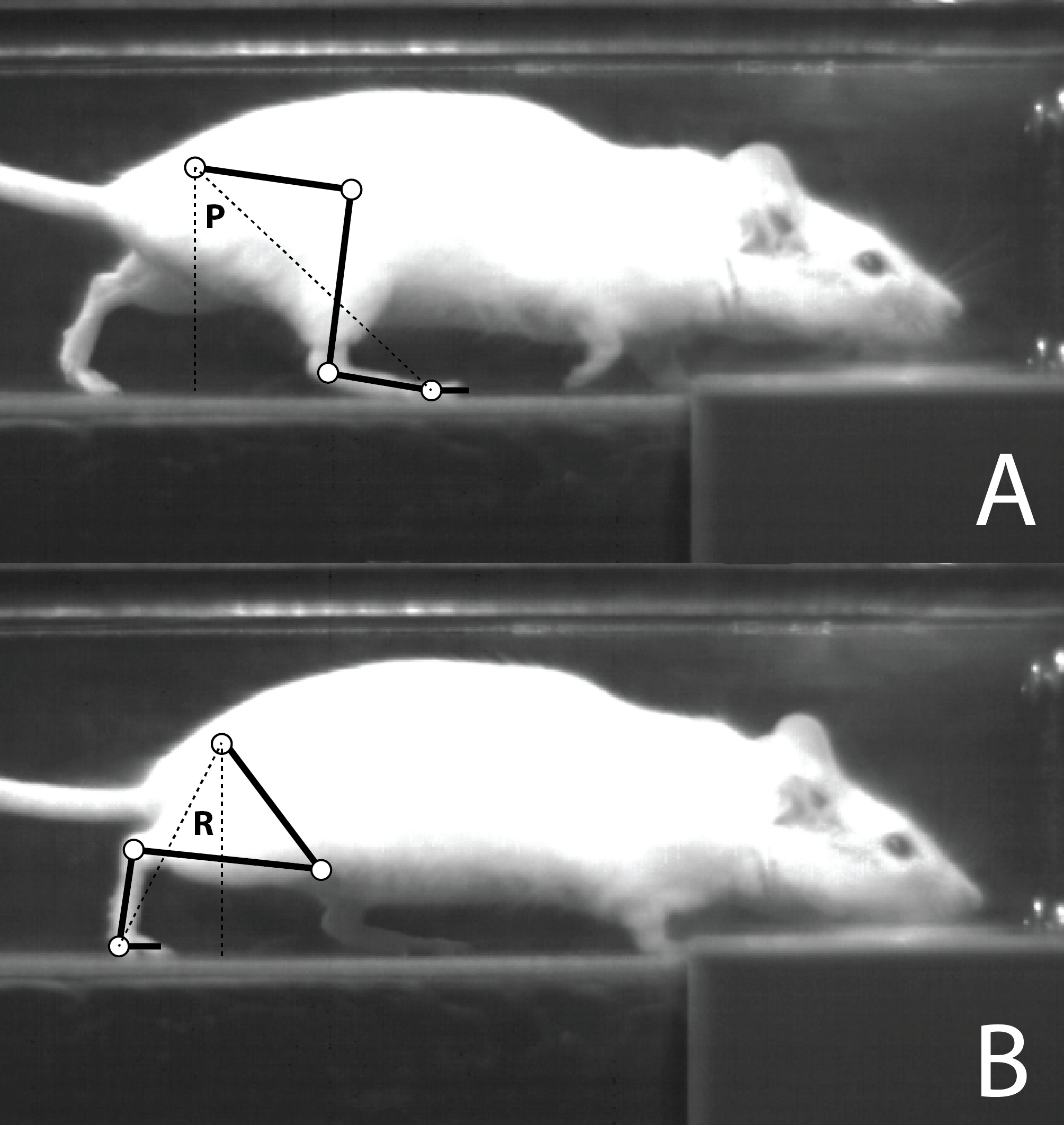

Supplement: Figure S1 — Sample video footage of a mouse running on a treadmill, showing the frame at the initiation of stance for the right hind paw (A, touch-down), and the last frame before the initiation of the swing phase for the same limb (B, toe-off). A stick model of the right hind limb is superimposed on each frame, showing the approximate location of the hip, knee, ankle and metatarsophalangeal joints (white circles), as well as the femur, tibia, tarso-metatarsus and forefoot (phalanges) (black lines). This model was used to estimate the protraction angle at touch down (P), and the retraction angle at toe-off (R). The sum of the protraction and retraction angles is the excursion angle. [file peerj-05-3008-s002.png]
