# Supplementary material for: Gait changes in a line of mice artificially selected for longer limbs
Source: PeerJ. 2017 Feb 22;5:e3008. doi: 10.7717/peerj.3008 (PMC5324776; doi:10.7717/peerj.3008)
Supplement: Table S1 — Comparison of limb excursion angles during stance in Control and Longshanks mice. Data reported as means ±SEM (in degrees), in a sample of 10 mice unrelated to the mice used in this study. The angle values for each individual were based on 4–5 steps. No significant differences were found between the lines in any of the angles (two-tailed t-tests, 0.13 < p < 0.83). [file peerj-05-3008-s003.docx]

**Table S1: Comparison of limb excursion angles during stance in Control and Longshanks mice.** Data reported as means ± SEM (in degrees), in a small sample of 10 mice unrelated to the mice used in this study. The angle values for each individual were based on 4-5 steps. No significant differences were found between the lines in any of the angles (two-tailed t-tests, 0.13<p<0.83).

| **Line** | **Protraction angle (°)** | **Retraction angle (°)** | **Excursion angle (°)** |
| --- | --- | --- | --- |
| Control (n=5) | 45.9 (2.8) | 25.8 (1.2) | 71.8 (2.4) |
| Longshanks (n=5) | 52.6 (2.8) | 25.0 (3.6) | 77.6 (5.1) |
| Pooled (n=10) | 49.2 (2.2) | 25.5 (1.8) | 74.7 (2.8) |
